# Towards sustainable lipidomics: computational screening and experimental validation of chloroform-free alternatives for lipid extraction

**DOI:** 10.1007/s00216-025-06136-z

**Published:** 2025-10-04

**Authors:** Andrea Venturi, Michele Wölk, Sider Penkov, Gabriele Cruciani, Maria Fedorova, Laura Goracci

**Affiliations:** 1 DAISY Lab (Drug Discovery-Artificial Intelligence-Organic Synthesis), Department of Chemistry, Biology and Biotechnology, University of Perugia, Via Elce di Sotto 8, Perugia, 06123 Italy; 2 Center of Membrane Biochemistry and Lipid Research, University Hospital and Faculty of Medicine Carl Gustav Carus of TU Dresden, Dresden, Germany

**Keywords:** Lipidomics, Lipid extraction, Green solvents, Chloroform alternatives, Modelling

## Abstract

Sample preparation, particularly lipid extraction, plays a critical role in lipidomics workflows and strongly influences analytical outcomes. In biomedical research, the most commonly used lipid extraction protocols rely on chloroform due to its favorable physicochemical properties, including the ability to dissolve both polar and apolar lipids, as well as its high volatility. Although well-established chloroform-based methods enable high recovery of a broad range of lipids, concerns about the toxicity and environmental impact of chloroform necessitate the development of more sustainable alternatives. In this study, a combined computational and experimental strategy was employed to identify and validate greener solvents suitable for lipid extraction in lipidomics applications. Solvent selection was guided by Hansen solubility parameters, Abraham solvation descriptors, and principal component analysis, yielding five candidate solvents as potential chloroform alternatives. Evaluation of solvent sustainability and human health risk was conducted using CHEM21 criteria and supplemented by a comprehensive literature review. Initial validation of extraction efficiency for identified candidates was performed using synthetic lipid standards in the absence of a biological matrix, followed by testing lipid extraction from human plasma using both monophasic and biphasic extraction protocols. The highest extraction efficiency was achieved using a single-phase extraction method based on cyclopentyl methyl ether, which exhibited comparable and even superior performance to the conventional chloroform-based Folch protocol. Overall, this study highlights the feasibility of replacing chloroform with less hazardous solvents in lipidomics workflows without compromising analytical performance, and provides a comparative assessment of computational solvent prediction approaches for green chemistry applications.

**Graphical Abstract:**

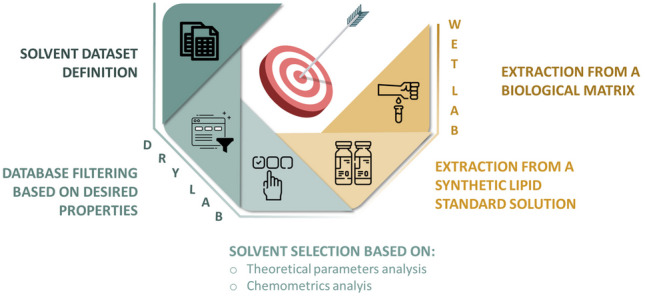

**Supplementary Information:**

The online version contains supplementary material available at 10.1007/s00216-025-06136-z.

## Introduction

Alterations in the cellular and tissue lipidome are closely linked to the onset and progression of numerous human diseases [1–5]. As a result, lipidomics has emerged as a distinct sub-discipline of analytical chemistry dedicated to the large-scale study of lipidomes, integrating biological, biochemical, and computational methodologies [6]. Currently, mass spectrometry (MS) represents the core analytical platform for lipid profiling [7]. A typical lipidomics workflow encompasses several key steps: sample collection and storage, sample preparation (including lipid extraction, cleanup, and solvent concentration), (liquid chromatography, LC-)MS analysis, data processing, statistical evaluation, and biological interpretation [8]. Given the immense structural and physicochemical diversity of lipid species—ranging from highly polar oxylipins to strongly hydrophobic triglycerides—comprehensive lipid extraction remains a major analytical challenge [9]. No single solvent or solvent mixture can efficiently extract the full spectrum of lipid classes due to their wide polarity range [9]. Predicted LogP values for lipid species span approximately 40 orders of magnitude on the *n*-octanol/water partition coefficient scale. Acylcarnitines represent some of the most polar lipid classes (estimated LogP between −5 and 5), whereas triglycerides are among the least polar, with LogP values ranging from 30 to 35 [9]. While such estimates may vary depending on the computational tools employed, they illustrate the exceptionally broad chemical space encompassed by lipidomes. Consequently, sample preparation—particularly the extraction step—is critical in lipidomics workflows. Many complex lipids are integral components of biological membranes and interact with matrix constituents such as proteins [10] and glycans [11, 12] via non-covalent interactions, including hydrophobic forces, van der Waals interactions, hydrogen bonding, and ionic interactions. Effective extraction therefore requires solvents capable not only of solubilizing a diverse array of lipids but also of disrupting these intermolecular associations [13]. Highly polar solvents with elevated dielectric constants, such as methanol, are particularly effective at breaking hydrogen bonds and ion-dipole interactions between lipids and proteins [14]. Other components of solvent mixtures contribute primarily to lipid solubilization, driven by hydrophobic and polar interactions. Beyond solubility considerations, solvent selection is also influenced by physicochemical and practical factors, including volatility (to facilitate evaporation post-extraction), chemical inertness (to prevent artifact formation), compatibility with aqueous phases (for biphasic extractions), and cost-effectiveness [15].

Although highly effective, conventional lipid extraction methods commonly rely on hazardous organic solvents that pose long-term risks to both human health and the environment. Among these, chloroform remains a major concern, despite its well-established efficacy in dissolving lipids across a wide polarity range [13]. Its favorable physicochemical properties—including good volatility (boiling point: 61 °C), biphasic behavior with aqueous solvents, and low cost—have contributed to its widespread use in lipid extraction protocols over the past decades [16–20]. However, the significant health hazards associated with chloroform exposure [21], along with its environmental toxicity [22, 23], have led to increased scrutiny. As a result, numerous pharmaceutical companies and sustainability initiatives—such as the ACS GCI Pharmaceutical Roundtable [24], GSK [25], Pfizer [26], Sanofi [27], and CHEM21 [28]—have recommended restricting or eliminating its use. These concerns have created a pressing need to identify more sustainable, yet analytically effective, alternatives to chloroform in lipidomics workflows.

The aim of this study was to identify environmentally and human health-friendly solvents capable of replacing chloroform in established lipid extraction protocols. Candidate solvents were selected through a combination of *in silico* approaches, including evaluation of Hansen solubility parameters [29], Abraham solvation descriptors [30], and principal component analysis (PCA) based on physicochemical properties. An initial assessment of Safety, Health, and Environmental (SHE) risks was conducted using the solvent classification framework proposed by the CHEM21 consortium [28], and further extended with additional literature-based evaluations for the most promising candidates. The extraction performance of the selected solvents was experimentally validated using both a synthetic lipid standard mixture and human plasma. This study not only demonstrates the applicability of computational solvent selection methods in lipidomics but also highlights the potential of such approaches to identify sustainable solvents with favorable analytical performance.

## Materials and methods

### Principal component analysis (PCA)

Principal component analysis (PCA) was performed using Lipostar 2 software [31]. A subset of 83 solvents from the filtered Diorazio’s dataset [32] was used as the object set. Twenty-nine physicochemical descriptors from the same dataset were selected as variables (see Results and Discussion section for details)**.** Data were imported as.csv format, and autoscaling was applied prior to analysis. The NIPALS algorithm [33] was used for missing values imputation.

### Chemicals, samples, and lipid standards

EquiSPLASH LIPIDOMIX Mass Spec Standard and SPLASH LIPIDOMIX Mass Spec Standard were purchased from Avanti Polar Lipids Inc. (Alabaster, AL, USA). Butylated hydroxytoluene (BHT; reagent grade ≥ 99%), (*Z*/*E*)−1,2-dichloroethene (DCE; ≥ 98%), dichloromethane (DCM; 98%), *iso*-butyl acetate (iBuAc; 98%), 2-methyltetrahydrofuran (2-MeTHF; EMPLURA®, ≥ 99%), cyclopentyl methyl ether (CPME; ≥ 99%), ammonium formate (NH_4_HCO_2_, LC-MS grade, ≥ 99.0%), formic acid (HCOOH; ≥ 98%), acetonitrile (CH_3_CN; ≥ 99.9%), chloroform (CHCl_3_; ≥ 99.8%), methanol (MeOH; ≥ 99.9%), and methyl *tert*-butyl ether (MTBE; ≥ 99.8%) were purchased from Merck (Burlington, MA, USA). Isopropanol (*i*-PrOH) and water (H_2_O) were purchased from Biosolve (Dieuze, Lorraine, FR; all ULC-MS grade, ≥ 99.95%). Human blood plasma was purchased from Merck (Burlington, MA, USA); the lyophilized powder was reconstituted in Milli-Q water according to the manufacturer's instructions.

### Lipid extraction

Extraction of synthetic lipid standards (EquiSPLASH LIPIDOMIX) was performed following the methanol–methyl *tert*-butyl ether-chloroform (MMC) protocol [18], substituted by each candidate solvent while maintaining the original volume ratio. Briefly, EquiSPLASH LIPIDOMIX solution (10 µL; *n* = 6) was added to the ice-cold H_2_O (40 µL). A total of 500 µL of solvent mixture (MeOH/MTBE/X, 1.33:1:1, v/v/v; where X represents either chloroform or a selected alternative solvent) was added to the sample. The mixture was vortexed for 30 s and subsequently shaken for 60 min at 1000 rpm at 4 °C. Following centrifugation (10 min, 20,000 × g, 4 °C), 450 µL of the supernatant was collected, evaporated under a gentle nitrogen stream, and reconstituted in 450 µL of *i*-PrOH/H_2_O (7:3, v/v). An aliquot of 200 µL was transferred to an autosampler vial containing a glass insert for UHPLC-MS analysis.

For lipid extraction from human blood plasma, butylated hydroxytoluene (BHT, 0.01% w/v) was added to each solvent mixture to prevent lipid oxidation. Lipid extraction from reconstituted plasma (5 µL) was performed using MTBE [34], Folch [16], and MMC [18] protocols, employing either traditional chloroform-based mixtures or alternative solvents wherein chloroform was replaced by equal volumes of cyclopentyl methyl ether (CPME), 2-methyltetrahydrofuran (2-MeTHF), or *iso*-butyl acetate (iBuAc). Notably, 2-MeTHF could not be used in the Folch protocol due to failure to achieve phase separation.

Lipid extraction according to the MTBE method [34] was performed as follows: to 5 µL of human blood plasma (*n* = 6), 180 µL of ice-cold MeOH was added and the sample vortexed for 30 s. Subsequently, 600µL of ice-cold MTBE was added, followed by vortexing for 30 s and incubation on a rotary shaker for 60 min at 40 rpm and 4 °C. Phase separation was induced by adding 150 µL of ice-cold H_2_O, vortexing for 30 s, and further incubation on a rotary shaker for 10 min at 40 rpm and 4 °C. Samples were then centrifuged for 10 min at 1000 × g and 4 °C. The upper organic phase (540 µL) was carefully collected into a new 1.5-mL Eppendorf tube and dried using a rotary vacuum concentrator (RVC 2-25 CDplus, Martin Christ GmbH). Dried lipid extracts were reconstituted in 50 µL of *i*-PrOH, shaken for 15 min, and centrifuged for 5 min at 10,000 × g and 4 °C. Finally, 40 µL of the supernatant was transferred into autosampler vials for subsequent UHPLC-MS analysis.

For extraction based on the Folch method [16], 5 µL of human blood plasma (*n* = 6) was mixed with 300 µL of ice-cold MeOH and vortexed for 30 s. Subsequently, 600 µL of ice-cold solvent X (X = chloroform, CPME, or iBuAc) was added, followed by vortexing for 30 s and incubation on a rotary shaker for 60 min at 40 rpm and 4 °C. Phase separation was induced by adding 150 µL of ice-cold H_2_O, vortexing for 30 s, and further incubation on a rotary shaker for 10 min at 40 rpm and 4 °C. After centrifugation (10 min, 1000 × g, 4 °C), the following volumes of phase were collected into new 1.5-mL Eppendorf tubes: (i) 540 µL of the upper phase for CPME; (ii) 565 µL of the upper phase for iBuAc; and (iii) 585 µL of the lower phase for chloroform. Samples were dried using a rotary vacuum concentrator. The dried lipid extracts were reconstituted in 50 µL of *i*-PrOH, shaken for 15 min, and centrifuged (5 min, 10,000 × g, 4 °C). Finally, 40 µL of the supernatant was transferred into autosampler vials for subsequent UHPLC-MS analysis.

Lipid extraction following MMC method [18] from 5 µL of human blood plasma (*n* = 6) was performed as described above. Dried lipid extracts were reconstituted in 50 μL of *i*-PrOH, shaken for 15 min and centrifuged (5 min, 10,000 × g, 4 °C). 40 µL of the supernatant was transferred into autosampler vials for subsequent UHPLC-MS analysis.

Extraction recovery was assessed using synthetic isotopically labeled lipid standards (EquiSPLASH or SPLASH LIPIDOMIX) spiked into the samples before (*n* = 3) or after extraction (*n* = 3). Recovery (%) was calculated as the ratio of the average peak areas of the monoisotopic ion of the analyte in samples spiked prior to extraction to those spiked post-extraction, multiplied by 100, according to the following equation:

$$R_{\mathit x}\mathit{\left(\%\right)}\mathit=\frac{{\mathit A}_{\mathit x}\mathit\;\mathit(\mathit s\mathit p\mathit i\mathit k\mathit e\mathit\;\mathit p\mathit r\mathit e\mathit\;\mathit-\mathit\;\mathit e\mathit x\mathit t\mathit r\mathit a\mathit c\mathit t\mathit i\mathit o\mathit n\mathit)}{{\mathit A}_{\mathit x}\mathit\;\mathit(\mathit s\mathit p\mathit i\mathit k\mathit e\mathit\;\mathit p\mathit o\mathit s\mathit t\mathit-\mathit\;\mathit e\mathit x\mathit t\mathit r\mathit a\mathit c\mathit t\mathit i\mathit o\mathit n\mathit)}\times100$$

where *R*_*x*_ represents the percentage recovery of lipid *x*, and *A*_*x*_ denotes the monoisotopic peak area of lipid *x*.

The use of the SPLASH LIPIDOMIX internal standard mixture, encompassing a broad concentration range (1 ng/μL for monoacylglycerol MG 18:1[2]H7 to 178 ng/μL for cholesteryl ester CE 18:1[2]H7; Table S1), enabled simultaneous assessment of recovery across lipid species spanning low, medium, and high abundance.

### UHPLC-MS/MS analysis

RPLC and MS acquisition parameters are provided in Supplementary information.

## Results and discussion

### Definition of the solvent dataset

**Fig. 1:**
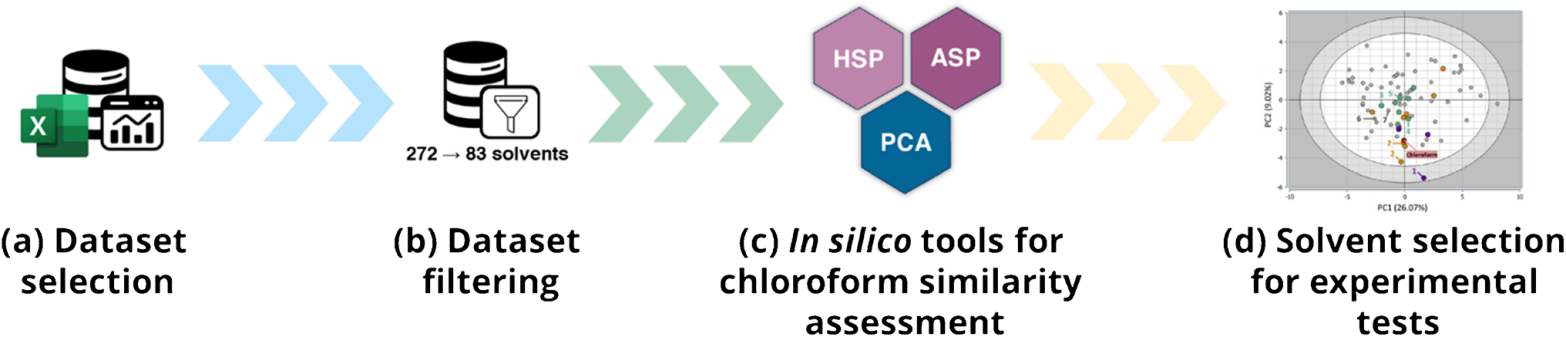
Schematic overview of the *in silico* workflow for computational selection of sustainable solvents as alternatives to chloroform. The initial dataset compiled by Diorazio et al., consisting of 272 solvents characterized by 80 variables (**a**), was filtered to exclude solvents with undesirable properties, reducing the set to 83 solvents (**b**). Three complementary approaches—Hansen solubility parameters (HSP), Abraham solvation parameters (ASP), and principal component analysis (PCA) based on selected physicochemical descriptors—were applied to identify solvents with similarities to chloroform (**c**). Integration and comparison of results from all three methods guided the final selection of candidate solvents for experimental validation (**d**)

The flowchart of the *in silico* procedure employed to select solvents with chloroform-like properties is presented in Fig. 1. The starting point was the solvent dataset compiled by Diorazio et al. [32], originally assembled by AstraZeneca. This dataset comprises 272 solvents commonly used in pharmaceutical processes and research, characterized by 80 variables encompassing classifications, scores, and physicochemical properties (Table S2). This initial dataset was filtered based on two criteria. First, solvents were classified as “recommended”, “problematic”, or “hazardous” according to their environmental, health, and safety impact, following the CHEM21 consortium classification [28]. All solvents ranked as “hazardous” were excluded except for those routinely used in lipid extraction (i.e., chloroform, dichloromethane, methanol, *n*-hexane, and methyl *tert*-butyl ether (MTBE)) to serve as reference compounds. Second, solvents with boiling points above 125 °C were removed to minimize evaporation time after lipid extraction. After filtering, the dataset was reduced from 272 to 83 solvents (Table S3), which were then used in computational models to select chloroform-like alternatives.

In recent years, numerous computational tools have been developed to facilitate solvent selection, providing efficient alternatives to traditional trial-and-error methods that are often time-consuming and resource-intensive. Conventional strategies typically compare macroscopic physical properties, such as dipole moment, to qualitatively or quantitatively assess solvent effects. However, this approach is limited, as solute-solvent interactions occur at the molecular scale. Solubility parameters represent another common strategy in solvent replacement. Among these, Hansen solubility parameters (HSP) [29], which generally describe solute-solvent interactions within a three-dimensional chemical space defined by dispersion, polar, and hydrogen bonding components, have been successfully applied to solvent selection in lipidomics [35–37]. In this model, two molecules positioned in close proximity within HSP space—reflected by a smaller HSP distance (*R*_a_)—are more likely to be mutually soluble, indicating similar solubility properties. An alternative theoretical model is provided by the Abraham solvation parameters (ASP) [30], which represent a linear free-energy relationship based on the cavity model of solvation. In this approach, the average difference (Δ) between individual Abraham descriptors of a two solvents is calculated, with smaller Δ values indicating greater similarity and, therefore, a higher likelihood of solvation. Although ASP has been successfully applied in various fields [38–40], to our knowledge, it has not yet been employed in lipidomics. More detailed descriptions of the HSP and ASP models are provided in the Supplementary Information. Additionally, unsupervised multivariate techniques such as principal component analysis (PCA) offer a data-driven approach to solvent selection. By visualizing solvents in the chemical space defined by principal components, structurally or functionally similar solvents cluster together, enabling the identification of candidates with comparable physicochemical properties.

In this study, HSP, ASP, and PCA models were evaluated to identify candidate solvents for chloroform replacement in lipid extraction using Diorazio’s dataset supplemented with data from the literature [41, 42] (Fig. 1; Tables S4 and S5). In the HSP approach, guided by previous studies indicating favorable solvent selection with *R*_a_ values between 4 and 6 [43–45], 12 candidates with *R*_a_ < 5 were selected. Similarly, for the ASP model, twelve solvents with the smallest average Δ were chosen. Interestingly, the solvent sets identified by the HSP and ASP models were largely distinct (Table 1), reflecting their differing theoretical foundations. Nevertheless, four solvents—dichloromethane (DCM), thiophene, tetrahydrofuran, and fluorobenzene—were identified by both approaches.

**Table 1:** Twelve top-ranked solvents selected based on their similarity to chloroform, as determined by Hansen solubility parameters (HSP, left) or Abraham solvation parameters (ASP, right)

Top-ranked solvents by HSP		Top-ranked solvents by ASP	
Solvent	***R***_a_	Solvent	Average Δ
1,1,2-Trichloroethane	2.59	(*E*)−1,2-Dichloroethene (DCE)	0.039
Cyclopentyl methyl ether (CPME)	2.87	(*Z*)−1,2-Dichloroethene (DCE)	0.051
2-Methyltetrahydrofuran (2-MeTHF)	2.97	1,1-Dichloroethane	0.091
Piperidine	3.03	Fluorobenzene	0.140
Thiophene	3.12	Perfluorobenzene	0.143
Dichloromethane (DCM)	3.32	Dichloromethane (DCM)	0.147
Pyrrolidine	3.81	Tetrahydrofuran	0.165
Tetrahydrothiophene	3.95	Thiophene	0.173
Tetrahydrofuran	4.01	Propyl formate	0.180
Toluene	4.09	*iso*-Butanol	0.181
Fluorobenzene	4.80	Nitroethane	0.182
*iso*-Butyl acetate (iBuAc)	4.81	Methyl propionate	0.187

**Fig. 2:**
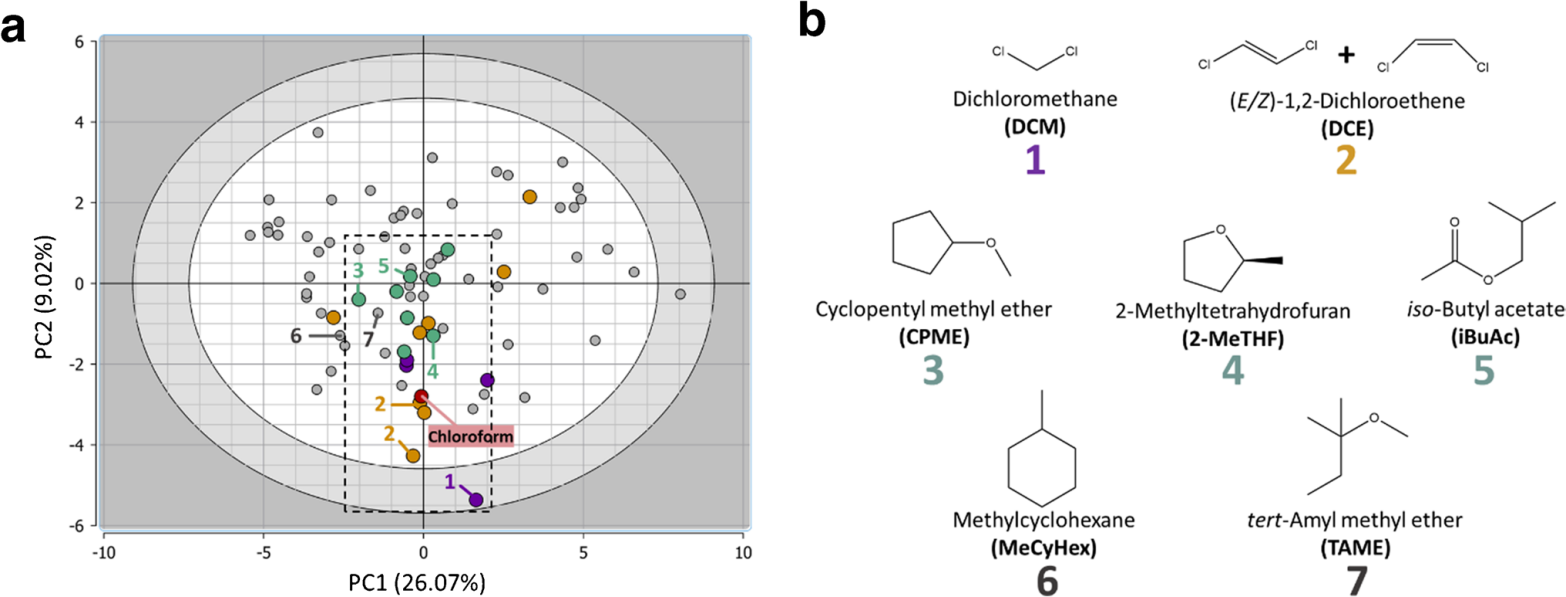
Final selection of solvents with chloroform-like properties. **a** PCA scores plot (PC1 vs PC2) generated using the 83 solvents from the filtered Diorazio et al. [32] dataset as objects and the block of 29 physicochemical descriptors from the original Diorazio’s paper as variables. The percentage of explained variance is reported in brackets. The results obtained from the Hansen solubility (HSP) and Abraham solvation (ASP) parameters were then evaluated altogether with the PCA by coloring the objects (solvents) in the PCA scores plot by the outcomes from HSP (green) and ASP (orange) approaches. Solvents that were top-ranked by both HSP and ASP approaches are shown in purple. Positions of negative controls are also labeled with numbers only. **b** Chemical structures of selected solvents used to test lipid extraction recovery

In the third approach, a PCA model was constructed using the 83 solvents as objects and 29 physicochemical descriptors from Diorazio’s dataset as variables (see Supplementary Table 2). The main advantage of using PCA in this context is its ability to place solvents prioritized by HSP and ASP models within unified chemical space, simultaneously accounting for multiple physicochemical properties. Indeed, nearly all chloroform alternatives selected by the HSP and/or ASP models were located in the central region of the PCA scores plot (Fig. 2a), exhibiting positions highly similar to chloroform along the first principal component (PC1). This component was primarily defined by variables related to H-bonding capacity, such as the Hansen H-bonding parameter, and solvatochromic α (hydrogen-bond donor) and β (hydrogen-bond acceptor) parameters on the right and mainly Abraham (V) descriptor (related to three-dimensional space occupied by the solute) on the left (see Supplementary Fig. 1 for the PCA loadings plot). In contrast, the same solvent candidates exhibited broader dispersion relative to chloroform along the second principal component (PC2), which was predominantly influenced on the one hand by lipophilicity (LogP_o/w_) and volatility (bottom region of the loadings plot) and on the other hand by boiling and flash points (upper region).

Based on these findings, solvents exhibiting a similar position to chloroform along PC1 but dispersed along PC2 were selected for experimental evaluation as potential substitutes for chloroform in lipid extraction protocols (Fig. 2b), following these criteria:

- 1. Prioritized by both HSP and ASP models
- 2. Prioritized by either HSP or ASP models
- 3. Not prioritized by either model (negative controls).

Additional considerations were applied during solvent selection, including practical constraints such as the exclusion of aromatic compounds due to benzene-like odor, as well as commercial availability and costs. Dichloromethane (DCM) was selected for experimental testing as it was prioritized by both the HSP and ASP models and represented the solvent most distant from chloroform along PC2. Both (*E*)- and (*Z*)−1,2-dichloroethene (DCE) were identified as the top candidates by the ASP model and, according to PCA, occupied a chemical space in very close proximity to chloroform. Since individual isomers were not commercially available, a mixture of *E/Z*-DCE was used in the experimental evaluation. Having already selected DCM and DCE, 1,1,2-trichloroethane—although the top-ranked solvent by the HSP model—was excluded to avoid an overrepresentation of chlorinated solvents. Instead, cyclopentyl methyl ether (CPME) and 2-methyltetrahydrofuran (2-MeTHF), the second and third highest-ranked HSP candidates, were selected. To further expand the chemical diversity of the test set, *iso*-butyl acetate (iBuAc) was included as one of the top HSP candidates, despite its more distant location from chloroform along PC2, but in a direction opposite to that of DCM. Finally, methylcyclohexane (MeCyHex) and *tert*-amyl methyl ether (TAME) were included as negative controls. Although both solvents occupied positions in PCA space similar to those of the candidates selected above, they were not prioritized by either the HSP or ASP models (see Supplementary Tables 3 and 4).

### Lipid extraction recovery using computationally selected solvents

Having defined the set of solvents for experimental evaluation (Fig. 2b), their lipid recovery performance was first assessed by using a mixture of synthetic lipid standards (EquiSPLASH LIPIDOMIX) in the absence of a biological matrix. The MMC method [18], a well-established one-phase extraction (OPE) protocol, was employed, with chloroform replaced by an equal volume of each candidate solvent identified through computational analysis. Lipid recovery values were compared to those obtained using the standard chloroform-based method (Table 2; Figure S2). All solvent candidates prioritized by HSP and/or ASP models exhibited recovery rates closely comparable to chloroform, with an average recovery of 95% across the lipid standard panel. In contrast, substantially lower recovery values—averaging 63%—were observed for methylcyclohexane (MeCyHex) and *tert*-amyl methyl ether (TAME), which were included as negative controls.

**Table 2:** Recovery values obtained for EquiSPLASH LIPIDOMIX lipid extraction (results are reported as values ± standard deviations)

Lipid	Chloroform	CPME	iBuAc	2-MeTHF	DCM	DCE	MeCyHex		TAME
LPC 18:1-[2]H7	**95 ± 2**	101 ± 1	99 ± 1	95 ± 2	93 ± 10	89 ± 9		66 ± 0	57 ± 3
LPE 18:1-[2]H7	**95 ± 1**	102 ± 3	95 ± 2	93 ± 3	92 ± 8	87 ± 14		56 ± 0	55 ± 3
PC 15:0/18:1-[2]H7	**96 ± 2**	99 ± 1	98 ± 1	97 ± 3	92 ± 4	91 ± 5		65 ± 4	56 ± 4
PE 15:0/18:1-[2]H7	**98 ± 3**	100 ± 2	106 ± 13	97 ± 6	92 ± 5	91 ± 9		57 ± 0	56 ± 3
PI 15:0/18:1-[2]H7	**99 ± 7**	110 ± 13	93 ± 10	94 ± 3	91 ± 1	83 ± 10		71 ± 1	61 ± 3
PS 15:0/18:1-[2]H7	**96 ± 6**	96 ± 11	101 ± 6	97 ± 2	93 ± 8	89 ± 5		64 ± 3	58 ± 5
PG 15:0/18:1-[2]H7	**99 ± 5**	103 ± 8	98 ± 2	95 ± 4	97 ± 11	88 ± 5		66 ± 4	56 ± 7
SM 18:1;O2/18:1-[2]H9	**96 ± 1**	100 ± 2	97 ± 3	94 ± 1	93 ± 5	93 ± 11		66 ± 1	57 ± 3
Cer 18:1-[2]H7;O2/15:0	**97 ± 1**	100 ± 4	98 ± 3	95 ± 4	97 ± 3	94 ± 8		74 ± 3	64 ± 5
MG 18:1-[2]H7/0:0/0:0	**97 ± 0**	100 ± 3	97 ± 3	92 ± 5	95 ± 5	91 ± 8		72 ± 4	66 ± 3
DG 15:0/18:1-[2]H7/0:0	**96 ± 4**	99 ± 1	97 ± 1	101 ± 5	93 ± 6	87 ± 4		76 ± 5	66 ± 5
TG 15:0/18:1-[2]H7/15:0	**95 ± 6**	97 ± 5	97 ± 4	97 ± 1	91 ± 6	92 ± 3		68 ± 4	62 ± 2
CE 18:1-[2]H7	**93 ± 8**	98 ± 5	99 ± 3	101 ± 20	90 ± 9	92 ± 6		56 ± 16	53 ± 7
Average	**96 ± 2**	**100 ± 3**	**98 ± 3**	**96 ± 3**	**93 ± 2**	**90 ± 3**		**66 ± 6**	**59 ± 4**

Having demonstrated the validity of the *in silico* selection strategy for identifying effective alternative solvents for lipid extraction, the list of candidate solvents was further refined by incorporating health and environmental risk considerations [46–49]. Although chlorinated solvents such as DCM and DCE showed good performance in extracting lipid standards, their continued use poses significant safety and environmental concerns. Indeed, similar to chloroform, they are flagged by the AstraZeneca Substance Avoidance Database (Table S2). Notably, in 2024, the United States Environmental Protection Agency (EPA) added DCM to the Toxic Substances Control Act (TSCA) list of substances subject to risk management, leading to a substantial reduction in its use across most settings, including laboratory research, due to its neurotoxic and carcinogenic properties [50].

In contrast, the remaining three selected solvents—CPME, iBuAc, and 2-MeTHF—are generally considered to pose lower risks to human health and the environment. CPME, in particular, is regarded as a relatively safe and sustainable solvent [51]. Although it is derived from a petrochemical precursor (cyclopentadiene), its synthesis is short, moderately energy-efficient, and proceeds via a 100% atom-economical reaction [52]. CPME is characterized by low toxicity, low peroxide formation (compared to other ethers), and a narrow explosion range. It is, however, classified as a highly flammable liquid and vapor and can cause skin and eye irritation [52]. Nevertheless, according to the European Chemicals Agency (ECHA) Guidance on Information Requirements and Chemical Safety Assessment, CPME is categorized as a low-hazard solvent [53].

iBuAc also stands out in terms of safety and environmental sustainability. It is typically synthesized via Fischer esterification from *iso*-butyl alcohol and acetic acid, both of which can be sourced from renewable feedstocks [54, 55]. iBuAc exhibits low toxicity and is neither irritating nor corrosive to the eyes or skin, with no major environmental concerns reported [56]. However, it remains a highly flammable liquid and vapor.

2-MeTHF is derived from renewable biomass sources such as corncobs or sugarcane bagasse. It is biodegradable, is non-toxic to the environment, and does not contribute to ozone depletion [57]. Although generally considered to have low toxicity [58], it is classified as harmful if swallowed, and it may cause skin irritation and serious eye damage. Additionally, it is a highly flammable liquid and vapor and has the potential to form explosive peroxides upon prolonged storage or exposure to air. While the selected alternative solvents are not without hazards, their associated risks are notably less severe than those of chloroform (Table 3). Based on these considerations, further lipid extraction experiments were conducted with CPME, iBuAc, and 2-MeTHF as alternative solvents.

**Table 3:** A summary of the key physicochemical and toxicological properties of evaluated solvents relevant to health and safety risk assessments

Substance	Primary acute hazards	Primary chronic hazards
Chloroform	Central nervous system (CNS) depression, respiratory irritation, gastrointestinal (GI) irritation, cardiac arrhythmias, delayed liver/kidney damage, skin/eye irritation	CNS damage (brain/nervous system), liver/kidney damage, cardiac arrhythmias
CPME	Skin/eye irritation, harmful if swallowed, nose/throat irritation,	Low sub-chronic toxicity, potential reproductive/developmental effects (at high doses)
2-MeTHF	Harmful if swallowed, skin irritation, eye damage, respiratory irritation	Airway disease, CNS effects
iBuAc	Respiratory tract irritation	Potential brain/nervous system damage

Next, the recovery of a synthetic lipid standards (SPLASH LIPIDOMIX) was evaluated in the presence of human blood plasma to assess solvent performance in a complex biological matrix. The alternative solvents—CPME, iBuAc, and 2-MeTHF—were tested using three established extraction protocols: the MMC method (one-phase extraction protocol, OPE), the Folch method (two-phase extraction protocol, TPE), and an MTBE-based protocol (a widely adopted chloroform-free TPE protocol). Notably, no phase separation was observed when 2-MeTHF was used in the Folch protocol, thereby restricting its application to the MMC method only. Lipid recovery was benchmarked against the corresponding chloroform-based protocols (Table 4; Figure S3).

**Table 4:** Recovery values of each lipid species contained in the SPLASH LIPIDOMIX obtained for the lipid extraction from human blood plasma (results are reported as values ± standard deviations)

Lipid	Folch			MTBE	MMC			
	Chloroform	CPME	iBuAc		Chloroform	CPME	iBuAc	2-MeTHF
LPC 18:1-[2]H7	**98 ± 1**	51 ± 5	41 ± 8	61 ± 1	114 ± 1	99 ± 6	90 ± 1	88 ± 8
LPE 18:1-[2]H7	**99 ± 1**	58 ± 1	50 ± 1	64 ± 1	100 ± 1	92 ± 1	90 ± 1	77 ± 1
PC 15:0/18:1-[2]H7	**100 ± 1**	77 ± 1	74 ± 3	82 ± 1	111 ± 1	92 ± 8	91 ± 2	92 ± 5
PE 15:0/18:1-[2]H7	**102 ± 1**	74 ± 3	75 ± 7	79 ± 1	104 ± 1	91 ± 1	95 ± 4	82 ± 5
PA 15:0/18:1-[2]H7	**78 ± 4**	83 ± 14	84 ± 6	82 ± 2	96 ± 8	105 ± 6	99 ± 21	94 ± 3
PI 15:0/18:1-[2]H7	**86 ± 1**	61 ± 2	60 ± 8	75 ± 4	99 ± 9	81 ± 2	104 ± 7	78 ± 2
PS 15:0/18:1-[2]H7	**83 ± 2**	86 ± 14	76 ± 5	81 ± 2	85 ± 6	85 ± 1	78 ± 1	62 ± 1
PG 15:0/18:1-[2]H7	**99 ± 1**	71 ± 3	68 ± 4	78 ± 1	108 ± 1	91 ± 2	89 ± 1	89 ± 2
SM 18:1;O2/18:1-[2]H9	**97 ± 1**	73 ± 1	68 ± 1	79 ± 1	108 ± 1	95 ± 1	89 ± 1	91 ± 1
Cholesterol-[2]H7	**85 ± 2**	105 ± 1	108 ± 1	95 ± 12	84 ± 11	82 ± 1	94 ± 1	92 ± 1
MG 18:1-[2]H7/0:0/0:0	**87 ± 2**	73 ± 1	73 ± 1	77 ± 3	102 ± 2	103 ± 1	82 ± 1	90 ± 1
DG 15:0/18:1-[2]H7/0:0	**93 ± 1**	81 ± 8	84 ± 10	82 ± 3	113 ± 2	95 ± 4	89 ± 5	88 ± 5
TG 15:0/18:1-[2]H7/15:0	**99 ± 2**	89 ± 7	94 ± 11	97 ± 3	111 ± 1	101 ± 2	94 ± 3	96 ± 3
CE 18:1-[2]H7	**102 ± 4**	86 ± 7	83 ± 8	78 ± 4	106 ± 4	102 ± 8	94 ± 6	90 ± 6
Average	**93 ± 8**	**76 ± 14**	**74 ± 17**	**79 ± 10**	**103 ± 9**	**94 ± 8**	**91 ± 6**	**86 ± 9**

Overall, chloroform-based Folch and MMC methods yielded comparable recoveries across most lipid classes, with the MMC protocol demonstrating superior performance for monoglyceride (MG), phosphatidylinositol (PI), and phosphatidic acid (PA) standards. When chloroform was replaced with CPME or iBuAc in the Folch protocol, a general decrease in recovery was observed. This reduction was particularly notable for lysophosphatidylcholine (LPC) and lysophosphatidylethanolamine (LPE), with recovery values decreasing by approximately 40% compared to the chloroform-based method (Table 4; Figure S3). In contrast, neutral lipid classes were less affected by the solvent substitution. Compared to the MTBE-based extraction protocol, neither CPME nor iBuAc alternatives in the Folch method provided improved recoveries. For instance, recovery rates of polar lipid standards such as LPE and PI were 20% and 15% lower, respectively, in the iBuAc-based Folch method relative to the MTBE protocol.

In the MMC protocol, the replacement of chloroform with CPME or iBuAc had minimal impact on lipid recovery, maintaining comparable values across most lipid classes. Only the use of 2-MeTHF led to a noticeable reduction in recovery, particularly for polar lipids such as LPE, PI, and phosphatidylserine (PS). Importantly, all chloroform-free MMC alternatives outperformed the MTBE-based method. In particular, the recovery of polar lipid species such as LPC and LPE was significantly higher when CPME or iBuAc was used in the MMC protocol, compared to the MTBE-based extraction (Table 4; Figure S3).

It is important to note that the SPLASH LIPIDOMIX mixture used here to assess extraction efficiency is specifically formulated as a labeled internal standard for human plasma lipidomics. It contains all major lipid classes at concentrations and relative ratios closely approximating physiological levels in human plasma. Within each lipid category, classes span a broad concentration range, classified as high (CE, cholesterol, PC), medium (TG, LPC, SM, PG), or low abundance (DG, PI, PA, LPE, PE, MG, PS). Extraction recovery values for lipid standards across these concentration levels showed no correlation with analyte abundance (Table S1). Instead, variations in recovery primarily reflected differences in lipid class chemistry rather than concentration-dependent effects.

Taken together, experimental validation illustrated that in the two-phase Folch extraction, the use of CPME and iBuAc consistently resulted in reduced lipid recoveries. A trend was observed between lipid polarity and the extent of recovery loss, with more pronounced reductions for highly polar species such as LPC and LPE. Conversely, in the one-phase MMC protocol, lipid polarity did not significantly influence recovery outcomes, and substitution of chloroform with CPME or iBuAc did not lead to significant loss in performance.

**Fig. 3:**
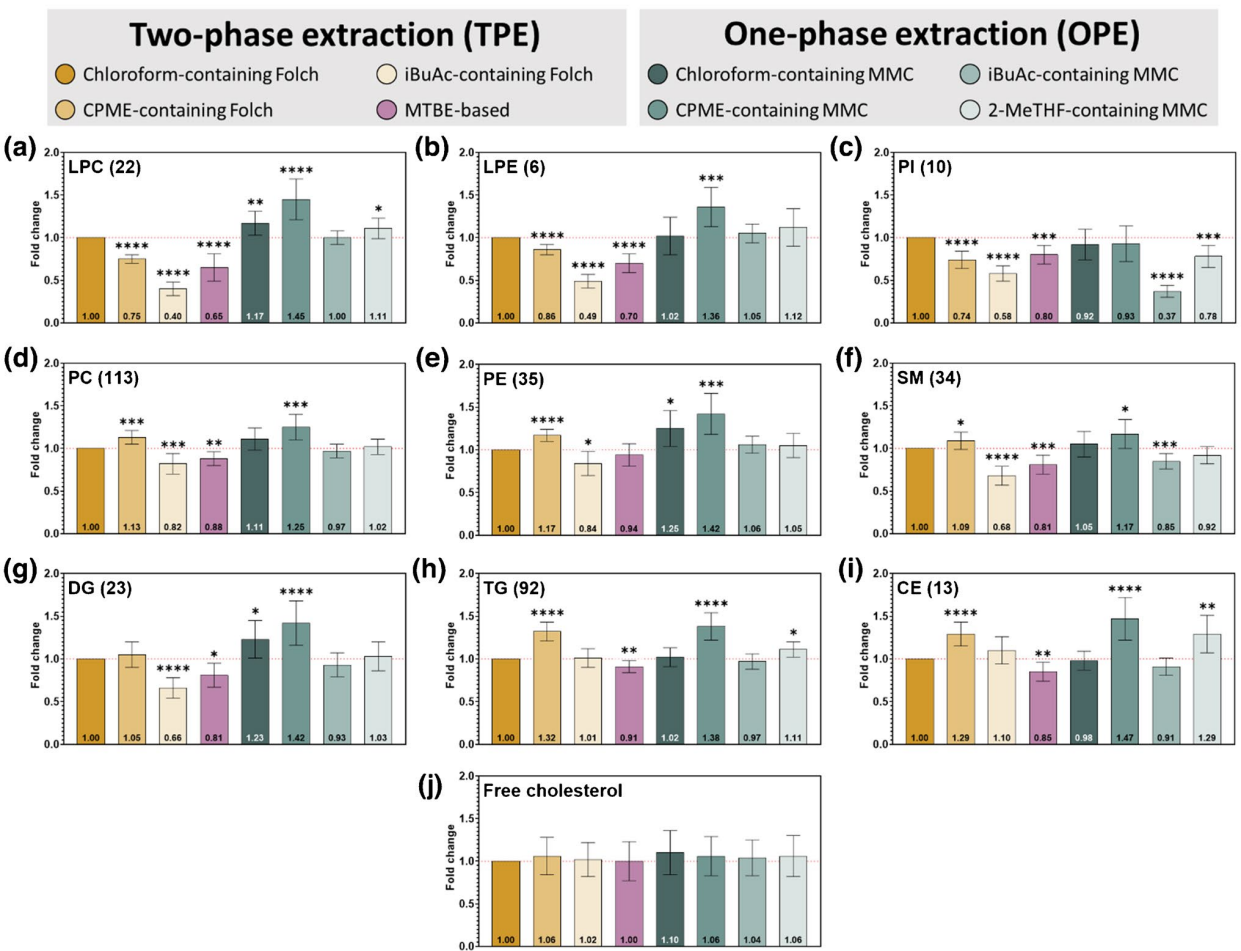
Extraction efficiencies of individual lipid classes using one-phase (OPE) and two-phase (TPE) extraction methods. For each extraction method and solvent system, peak areas of identified lipid species were first normalized to the corresponding deuterated internal standards from the SPLASH LIPIDOMIX mixture. Lipid extraction efficiency was then assessed based on the summed abundance of each lipid class from human blood plasma. Graphs depict fold changes (± standard deviation) relative to the chloroform-containing Folch protocol for: **a** lysophosphatidylcholine (LPC), **b** lysophosphatidylethanolamine (LPE), **c** phosphatidylinositol (PI), **d** phosphatidylcholine (PC), **e** phosphatidylethanolamine (PE), **f** sphingomyelin (SM), **g** diglycerides (DG), **h** triglycerides (TG), **i** cholesteryl esters (CE), and **j** free cholesterol. The number of integrated lipid species included in the analysis is indicated next to each lipid class label. Statistical significance relative to the chloroform-based Folch method was determined using Student’s *t*-test; significance levels are indicated above each bar: ****p < 0.001, ***p < 0.005, **p < 0.01, *p < 0.05. Bars without asterisks indicate no statistically significant difference

Finally, new extraction protocols were evaluated for the recovery of endogenous blood plasma lipids. Although over 380 individual lipid species from 19 different subclasses were annotated using Lipostar 2 software [31], only lipid classes matching those included in the SPLASH LIPIDOMIX mixture were considered for the analysis as due to the employed normalization strategy, which relied on the use of isotopically labeled internal standards. Thus, for each extraction method, the peak area of individual lipids was first normalized according to the peak area of the corresponding deuterated standard, and then lipid extraction efficiencies were assessed based on the summed class abundances of the human blood plasma lipids (Table S6). Lipid recovery was benchmarked against the chloroform-based Folch protocol (Fig. 3).

For more polar lipids, including LPC, LPE, and PI, chloroform-free two-phase extraction methods (CPME- and iBuAc-containing Folch, and MTBE) demonstrated significantly lower extraction efficiency compared to the traditional chloroform-based Folch method (Fig. 3a–c). Consistent with the results obtained for synthetic lipid standard recovery (Table 4), the most pronounced reduction in performance was observed for the iBuAc-containing Folch and MTBE protocols. While the CPME-containing Folch method showed marginally better performance among the chloroform-free TPE approaches, it still failed to reach the efficiency of the conventional chloroform-based extraction. In contrast, the application of OPE methods—employing CPME, iBuAc, or 2-MeTHF in the MMC protocol—resulted in extraction efficiencies for LPC, LPE, and PI that were comparable to, or in some instances exceeded, those obtained with the chloroform-containing Folch method. Notably, the CPME-based MMC protocol yielded the highest extraction efficiencies among all tested methods, with fold changes of 1.45, 1.36, and 0.93 for LPC, LPE, and PI, respectively.

The extraction of phospholipids of intermediate polarity—namely, phosphatidylcholines (PC), phosphatidylethanolamines (PE), and sphingomyelins (SM)—showed similar trends (Fig. 3d–f). Among the TPE methods, only the CPME-containing Folch protocol achieved better recovery compared to the chloroform-based Folch method. Importantly, the CPME-containing MMC method consistently outperformed chloroform-containing protocols, with fold increases of 1.25, 1.42, and 1.17 for PC, PE, and SM, respectively. In the case of diglycerides (DG, Fig. 3g), the highest recovery was achieved using the MMC extraction with CPME as well.

For neutral lipids, replacement of chloroform by CPME showed superior extraction efficiency both in TPE and OPE methods (Fig. 3h, i). Specifically, the replacement of chloroform with CPME led to enhanced extraction efficiency for triglycerides (TG) in both Folch and MMC protocols. For cholesteryl esters (CE), the CPME-based Folch and MMC methods also showed superior performance, with fold increases of 1.29 and 1.47, respectively, relative to the chloroform-based Folch protocol. In contrast, no statistically significant differences were observed between methods for free cholesterol, indicating that all protocols were equally effective for this lipid class.

Taken together, substitution of chloroform in the Folch protocol did not result in uniformly optimal performance across all lipid classes. While the CPME-containing Folch method showed promising results for lipids of medium and low polarity, the one-phase MMC methods using CPME consistently matched or exceeded the performance of the chloroform-based extractions across a broad range of lipid classes.

## Conclusion

This study identified CPME as a promising alternative to chloroform in commonly employed lipid extraction protocols, using a combined computational and experimental approach. While CPME has previously been proposed as a safer and more environmentally sustainable solvent [51], a more systematic validation within both one-phase and two-phase extraction protocols was conducted here. Furthermore, the study enabled critical evaluation of solvent selection strategies based on Hansen solubility parameters, Abraham solvation parameters, and a principal component analysis model constructed from physicochemical properties. Although physicochemical descriptors are routinely used to estimate solvent similarity, our findings suggest that the PCA-based selection strategy when not combined with HSP and ASP models may lead to false positives. For instance, MeCyHex and TAME, although located near CPME within the PCA-derived chemical space and falling within the region of interest identified in this study, were not prioritized by either HSP or ASP-based selection models and in practice demonstrated poor extraction performance.

Using human blood plasma as a model of a complex biological lipidome, the one-phase MMC method employing CPME demonstrated the highest extraction efficiency across a broad range of lipid classes. In contrast, the substitution of chloroform in the two-phase Folch method revealed pronounced solvent-lipid class selectivity. Among the lipid classes investigated, polar lipids (LPC, LPE, and PI) were significantly more affected by the replacement of chloroform with CPME or iBuAc, exhibiting notably reduced extraction efficiencies. This effect diminished with decreasing lipid polarity. Specifically, the CPME-containing Folch method showed improved performance over the chloroform-based method for medium-polarity lipids (e.g., PC, PE, SM) and neutral lipids (e.g., TG, CE). Interestingly, this solvent-dependent selectivity was not observed with the one-phase MMC method, for which no clear polarity-based trend was evident.

Analytically, the CPME-based MMC protocol appears to entail minimal trade-offs, offering substantial benefits in terms of operator safety and environmental compatibility. However, one practical limitation is the solvent cost: CPME is approximately 33% more expensive than chloroform. While the current application of this method remains limited to laboratory-scale workflows, its scalability could be significantly enhanced by robotic automation. The protocol itself presents no intrinsic barriers to scale-up, and future studies may focus on procedural refinements to facilitate integration with high-throughput platforms, potentially enabling its use in large-scale lipidomic or multi-omics studies.

In summary, this study proposes viable alternatives to chloroform-based lipid extraction, demonstrating the value of combining computational solvent screening with empirical validation to identify efficient, sustainable extraction strategies suitable for modern lipidomics.

## Supplementary Information

Below is the link to the electronic supplementary material.

Supplementary Material 1 (PDF 1.17 MB)

Supplementary Material 2 (XLSX 107 KB)

Supplementary Material 3 (XLSX 27.4 KB)

Supplementary Material 4 (XLSX 16.8 KB)

Supplementary Material 5 (XLSX 17.8 KB)

Supplementary Material 6 (XLSX 333 KB)

## Data Availability

Data are available from the authors by request.
