# Supplementary material for: Towards sustainable lipidomics: computational screening and experimental validation of chloroform-free alternatives for lipid extraction
Source: Anal Bioanal Chem. 2025 Oct 4;417(28):6451–62. doi: 10.1007/s00216-025-06136-z (PMC12596327; doi:10.1007/s00216-025-06136-z)
Supplement: Supplementary file 1 — Supplementary Material 1 (PDF 1.17 MB) [file 216_2025_6136_MOESM1_ESM.pdf]

Towards Sustainable Lipidomics: Computational Screening and Experimental Validation of Chloroform-Free Alternatives for Lipid Extraction

Andrea Venturi[1,2], Michele Wölk[2], Sider Penkov[2], Gabriele Cruciani[1], Maria Fedorova[2*], and Laura Goracci[1*]

[1] DAISY Lab (Drug Discovery-Artificial Intelligence-Organic Synthesis), Department of Chemistry, Biology and Biotechnology, University of Perugia, Via dell' Elce di Sotto 8, 06123 Perugia, Italy

[2] Center of Membrane Biochemistry and Lipid Research, University Hospital and Faculty of Medicine Carl Gustav Carus of TU Dresden, Dresden, Germany.

*Maria Fedorova, Lipid Metabolism: Analysis and Integration, Center of Membrane Biochemistry and Lipid Research, University Hospital Carl Gustav Carus and Faculty of Medicine of TU Dresden, Dresden, Germany; E-mail: maria.fedorova@tu-dresden.de

*Laura Goracci, DAISY Lab, Department of Chemistry, Biology and Biotechnology, University of Perugia, Perugia, Italy; E-mail: laura.goracci@unipg.it

Supplementary methods

UHPLC-MS/MS conditions

Synthetic lipid standards (EquiSPLASH LIPIDOMIX) were separated on a Thermo Dionex UltiMate 3000 series Liquid Chromatography system (Thermo Fisher Scientific, Waltham, MA, USA) equipped with an Accucore C18 column (150×2.1 mm; 2.6 µm, 150 Å – Thermo Fischer Scientific, Sunnyvale, USA). Lipids were separated by gradient elution with mobile phases A (CH_3CN/H_2O, 50/50, v/v) and B (*i*-PrOH/CH_3CN/H_2O, 85/10/5, v/v/v) both containing 0.1% of HCOOH and 5 mM NH_4HCO_2. Separation was performed at 45°C with a flow rate of 0.3 mL/min using the following gradient: 0.0-3.0 min – 20 to 40% B (curve 5), 3.0-16.0 min – 40 to 60% B (curve 5), 16.0-16.5 min – 60 to 70% B (curve 5), 16.5-24.0 min – 70 to 74% B, 24.0-28.0 min – 74 to 95% B (curve 5), 28.0-31.0 min – 95% isocratic, 31.0-31.1 – 95 to 20% B, 31.1-32.0 min – 20% isocratic. The autosampler temperature was maintained at 15 °C. the injection volume was 2 µL. LC was coupled online to quadrupole – orbitrap mass spectrometer (Q-Exactive, Thermo Scientific, San Jose, CA, USA) equipped with a heated electrospray ionization (HESI-II) source. Mass spectra were acquired in polarity switching ionization mode with the following ESI parameters: sheath gas – 50 a.u., auxiliary gas – 15 a.u., spray voltage – 3.5 kV (positive ion mode); - 2.5 kV (negative ion mode), ion transfer temperature – 320°C, S-lens RF level – 100% and aux gas heater temperature – 370°C. Data were acquired in full scan acquisition mode with a survey scan resolution 70000 (at *m/z* 200), AGC target 1e6, maximum IT 200 ms in a scan range of *m/z* 120-1800. All data were acquired in profile mode.

LC-MS/MS analysis of lipid extracts from human blood plasma were performed according to the method developed by Wölk and Fedorova [1]. Briefly, lipids were separated on a Vanquish Horizon UHPLC system (Thermo Fisher Scientific, Germering, Germany) equipped with an Accucore C30 column (150x2.1 mm; 2.6 mm, 150 Å - Thermo Fisher Scientific, Sunnyvale, USA). Lipids were separated by gradient elution with mobile phases A (CH_3CN/H_2O, 50/50, v/v) and B (*i*-PrOH/CH_3CN/H_2O, 85/10/5, v/v/v) both containing 0,1% of HCOOH and 5 mM NH_4HCO_2. Separation was performed at 50°C with a flow rate of 0.3 mL/min using the following gradient: 0.0-20.0 min – 10 to 80% B (curve 5), 20.0-24.0 min – 80 to 95% B (curve 5), 24.0-27.0 min – 95 to 100% B (curve 5), 27.0-32.0 min – 100% isocratic, 32.0-32.1 – 100 to 10% B, 32.1-40.0 min – 10% isocratic. The injection volumes were 2 µL (positive mode)/4 µL (negative mode). LC was coupled online to quadrupole – orbitrap mass spectrometer (Orbitrap Exploris 240 Mass Spectrometer - Thermo Scientific, San Jose, CA, USA) equipped with a HESI-II source. Mass spectra were acquired in positive and negative ionization modes with the following ESI parameters: sheath gas – 40 a.u., auxiliary gas – 10 a.u., sweep gas – 1 a.u., spray voltage – 3.5 kV (positive ion mode); -2.5 kV (negative ion mode), ion transfer temperature – 300°C, S-lens RF level – 35% and vaporizer temperature – 370°C. Data were acquired in data-dependent acquisition (DDA) modes with a survey scan resolution of 120 000 (at *m/z* 200), normalized AGC target 100%, maximum IT 100 ms in a scan range of *m/z* 200-1000. Data-dependent MS2 were acquired with a cycle time of 1.3 s witch a resolution of 15 000 at 200 m/z, normalized AGC target 100%, maximum IT 60 ms, isolation window 1.2 *m/z*, stepped normalized collision energies of 17, 27 and 37%, and dynamic exclusion 6s. All data were acquired in profile mode.

Hansen solubility parameters (HSP)

Hansen solubility parameters (HSP) [2] quantify solute-solvent interactions based on three key contributions: dispersion forces (δ_D), dipole moments (δ_P), and hydrogen bonds (δ_H). Hansen space is represented by the chemical space defined using these three coordinates. The closer two molecules located in Hansen space, the more likely they will dissolve in each other. The interaction radius (R_0; experimentally defined solubility space for each solute) and HSP distance (R_a) between two molecules (solute and solvent) are used to define the affinity of the solvent to the solute. R_a between solute *i* and solvent *j* depends on their respective solubility parameter components, as follows:

$$R_a = \sqrt{4(\delta_{Di} - \delta_{Dj})^2 + (\delta_{Pi} - \delta_{Pj})^2 + (\delta_{Hi} - \delta_{Hj})^2} \qquad \textbf{\textit{Eq.(1)}}$$

The affinity of the solvent to the solute is estimated based on the relative energy difference (RED), calculated as the ratio between R_a and R_0, considering that the lower the RED, the greater the affinity (RED ≤ 1 indicates high affinity, and RED > 1 indicates low affinity between a given solute and solvent).
In this study, the HSP reported in the Diorazio's dataset [3], or found in literature [4] were used. Chloroform has been assigned the role of the solute in the equation that calculates R_a for each solvent. Under this approach, a lower R_a value indicates a closer resemblance between the HSP of the solvent and those of chloroform. To evaluate similarity based on HSP only, R_a values were compared.

Abraham solvation parameters (ASP) model
Abraham solvation parameters (ASP) model [5] provides another effective approach for assisting in solvent selection. ASP model is a linear free-energy relationship that is used to describe the interactions between solutes and solvents for a given solute property and is based primarily on the parameterization of the cavity model of solvation [6]. In general, solute properties correlate with the six parameters listed below.
In this study, the ASP reported in the Diorazio's dataset [3], or found in literature [7] were used. The average differences between the ASP of chloroform and those of each solvent were calculated, which smaller difference indicating higher similarity to chloroform in terms of solvation properties.

Description of the parameters considered in the Abraham solvation parameters (ASP) model.

PARAMETER	DESCRIPTION
**E**	The excess molar refraction ((cm^3mol^{-1})/10) represents solute polarizability and gives a measure of the ability of a solute to interact with a solvent through n- and π-electron pairs.
**S**	The solute dipolar/polarizability parameter gives a measure of the solute's ability to stabilize a charge or dipole.
**A**	The hydrogen bond acidity descriptor measures the extent of hydrogen bonding by the solute in a basic solvent.
**B**	The hydrogen bond basicity descriptor measures the extent of hydrogen bonding by the solute in an acidic solvent.
**L**	The Ostwald solubility coefficient between gas to wet solvent at 298 K, represents cavity size and dispersion forces.
**V**	McGowan's characteristic volume ((cm^3mol^{-1})/100), used to describe the transfer between water and wet solvents, reflects the three-dimensional space occupied by the solute. It is calculated from the atomic size and the number of chemical bonds within the solute.

Supplementary Tables

Table S1. Quantities of lipids in the SPLASH LIPIDOMIX mixture employed in recovery experiments, alongside corresponding recovery rates obtained using chloroform-based and chloroform-free extraction protocols.

LIPID	Spiked concentration (ng/µL)	Folch			MTBE	MMC			
		$CHCl_3$	CPME	iBuAc		$CHCl_3$	CPME	iBuAc	2-MeTHF
CE 18:1-[2]H7	178.05	102±4	86±7	83±8	78±4	106±4	102±8	94±6	90±6
Cholesterol-[2]H7	49.2	85±2	105±1	108±1	95±12	84±11	82±1	94±1	92±1
PC 15:0/18:1-[2]H7	80.35	100±1	77±1	74±3	82±1	111±1	92±8	91±2	92±5
TG 15:0/18:1-[2]H7/15:0	28.65	99±2	89±7	94±11	97±3	111±1	101±2	94±3	96±3
LPC 18:1-[2]H7	12.75	98±1	51±5	41±8	61±1	114±1	99±6	90±1	88±8
SM 18:1;O2/18:1-[2]H9	15.45	97±1	73±1	68±1	79±1	108±1	95±1	89±1	91±1
PG 15:0/18:1-[2]H7	14.55	99±1	71±3	68±4	78±1	108±1	91±2	89±1	89±2
DG 15:0/18:1-[2]H7/0:0	4.7	93±1	81±8	84±10	82±3	113±2	95±4	89±5	88±5
PI 15:0/18:1-[2]H7	4.55	86±1	61±2	60±8	75±4	99±9	81±2	104±7	78±2
PA 15:0/18:1-[2]H7	3.7	78±4	83±14	84±6	82±2	96±8	105±6	99±21	94±3
LPE 18:1-[2]H7	2.65	99±1	58±1	50±1	64±1	100±1	92±1	90±1	77±1
PE 15:0/18:1-[2]H7	2.85	102±1	74±3	75±7	79±1	104±1	91±1	95±4	82±5
PS 15:0/18:1-[2]H7	2.1	83±2	86±14	76±5	81±2	85±6	85±1	78±1	62±1
MG 18:1-[2]H7/0:0/0:0	1	87±2	73±1	73±1	77±3	102±2	103±1	82±1	90±1

Table S2. List of 272 solvents commonly used in pharmaceutical processes and research, characterized by 80 variables encompassing classifications, scores, and physicochemical properties based on the original Diorazio's dataset.

Table S3. List of 83 solvents complimented by 29 physicochemical descriptors obtained from original Diorazio's dataset by filtering out solvents classified as "hazardous" according to CHEM21 consortium classification and solvents with the boiling point above 125 °C.

Table S4. Hansen solubility parameters (HSP) for the set of 83 solvents retrieved from the Diorazio's dataset and additional literature resource.

Table S5. Abraham solvation parameters (ASP) for the set of 83 solvents retrieved from the Diorazio's dataset and additional literature resource.

Table S6. Peak areas of lipids from human blood plasma obtained by analysing samples extracted with chloroform-based or chloroform-substituted protocols and normalized to the corresponding deuterated standards (SPLASH LIPIDOMIX). For each lipid class average sum and fold change relative to chloroform-based Folch method are provided.

Supplementary Figures

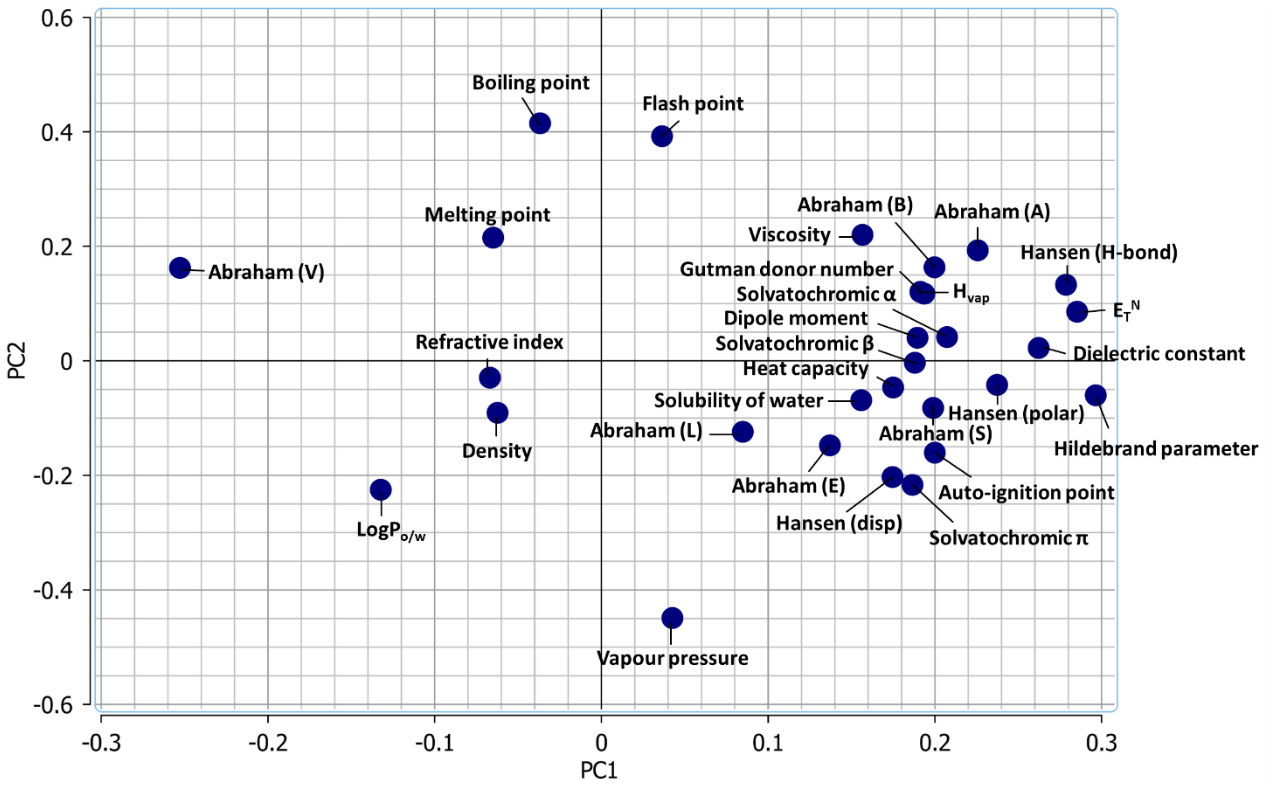

Figure S1. PCA loading plot (PC1 vs PC2) generated using the 83 solvents from filtered Diorazio et al. dataset as objects and the block of 29 physicochemical descriptors as variables.

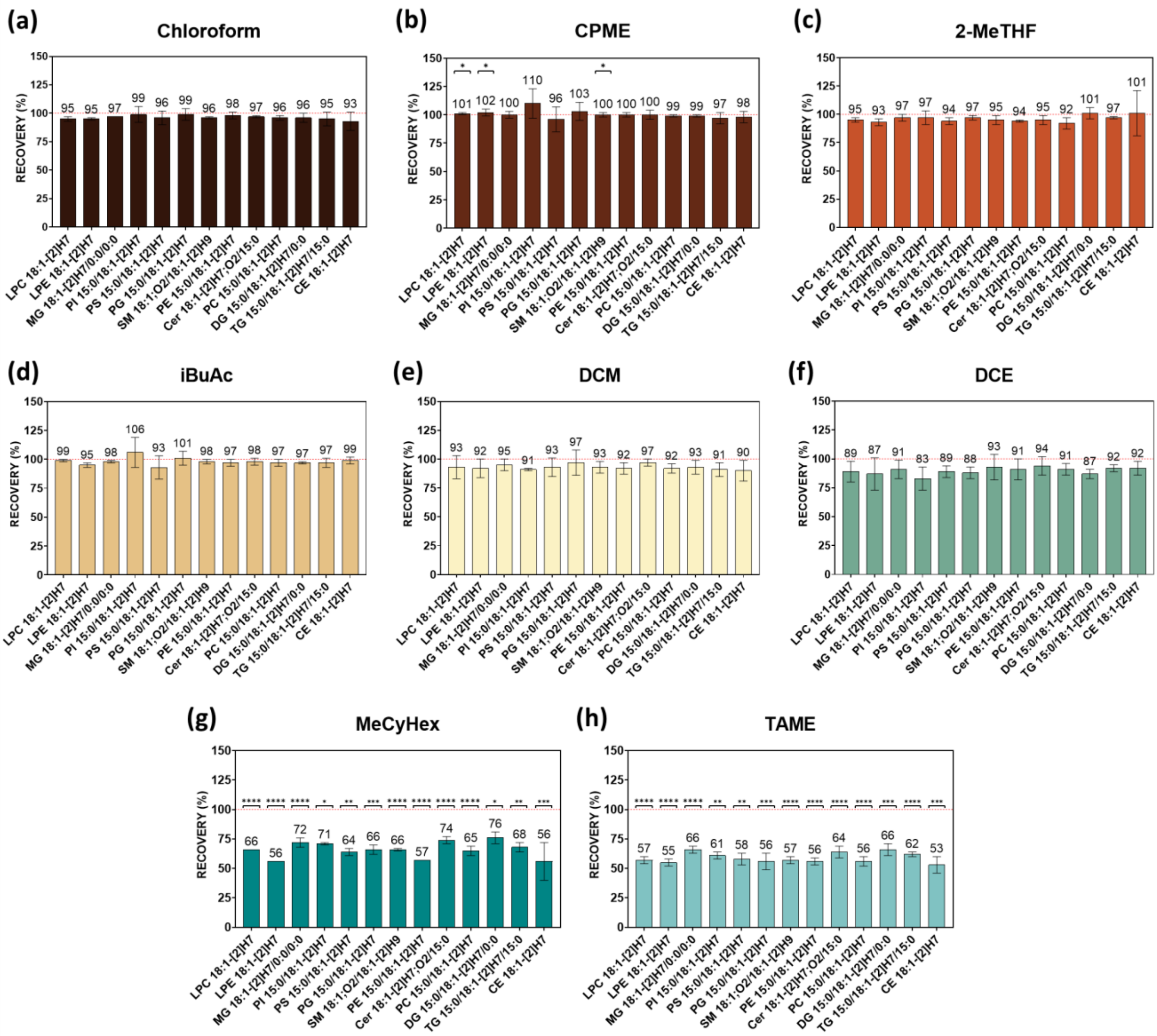

Figure S2. Recovery values obtained for lipid standards (EquiSPLASH LIPIDOMIX) using MMC protocol either with chloroform (a) or selected chloroform substitutes including (b) cyclopentyl methyl ether, CPME (b), 2-methyltetrahydrofurn, 2-MeTHF (c), *iso*-butyl acetate, iBuAc (d), dichloromethane, DCM (e), dichloroethene, DCE (f), methylcyclohexane, MeCyHex (g), and *tert*-amyl methyl ether, TAME (h). Recovery values (± standard deviation) were estimated as the ratio of the average areas of the monoisotopic peaks of the extracted lipid in the samples spiked before and post-extraction. Statistical significance relative to the chloroform-based MMC method was determined using Student's t-test; significance levels are indicated above each bar: **** = p-value < 0.001, *** = p-value < 0.005, ** = p-value < 0.01, * = p-value < 0.05.

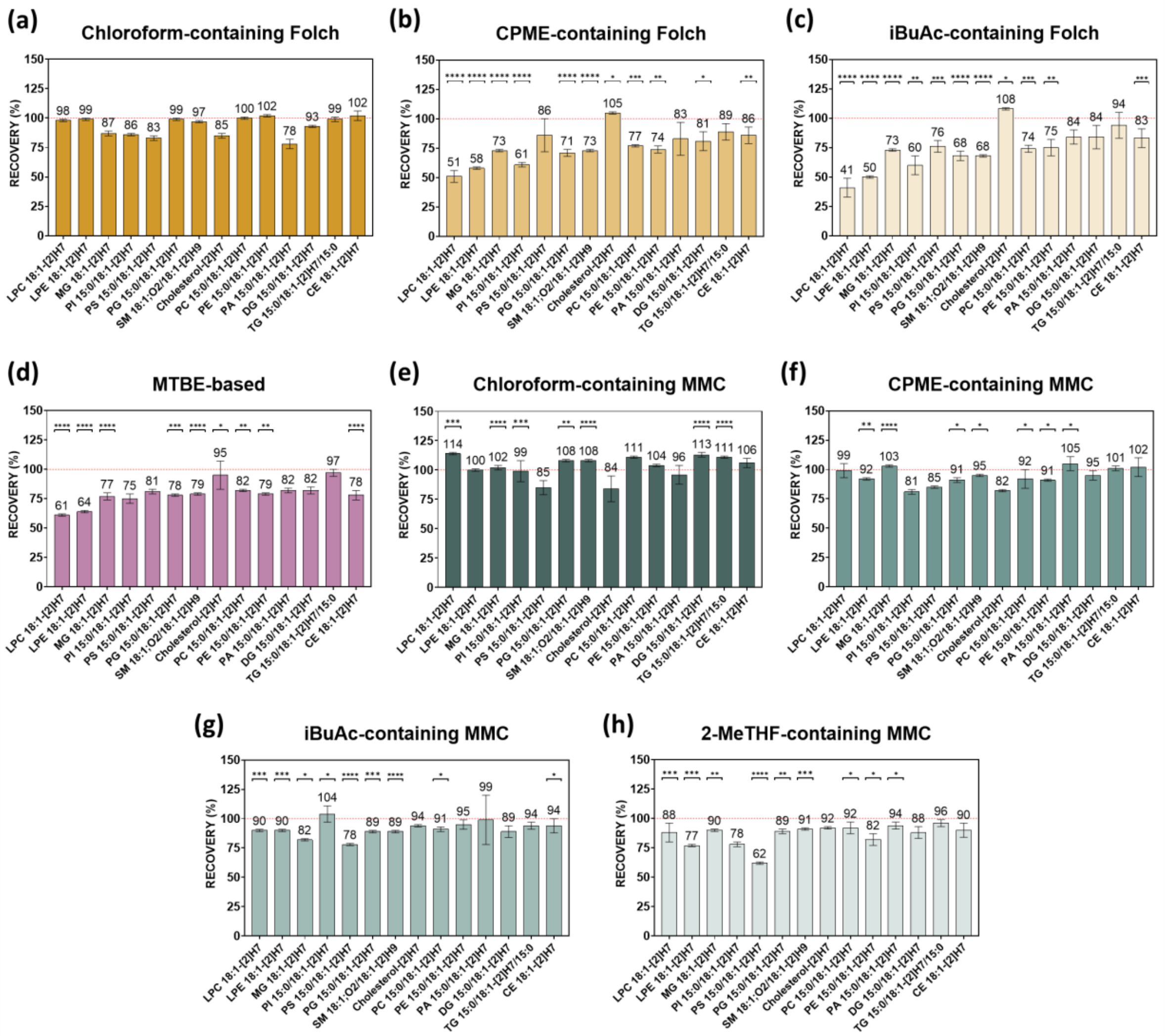

Figure S3. Recovery values obtained for the lipid standards (SPLASH LIPIDOMIX) for the extraction from human blood plasma using chloroform-based and chloroform-free protocols including traditional chloroform-containing Folch method (a), CPME-containing Folch method (b), iBuAc-containing Folch method (c), MTBE-based method (d), traditional chloroform-containing MMC method (e), CPME-containing MMC method (f), iBuAc-containing MMC method (g), and 2-MeTHF-containing MMC method (h). Recovery values (± standard deviation) were estimated as the ratio of the average areas of the monoisotopic peaks of the extracted lipid in the samples spiked before and post-extraction. Statistical significance relative to the chloroform-based MMC method was determined using Student's t-test; significance levels are indicated above each bar: **** = p-value < 0.001, *** = p-value < 0.005, ** = p-value < 0.01, * = p-value < 0.05.

References

1. Wölk M, Fedorova M (2025) Recommendations for Accurate Lipid Annotation and Semi-absolute Quantification from LC-MS/MS Datasets. In: Giera M, Sánchez-López E (eds) Clinical Metabolomics: Methods and Protocols. Springer US, pp 269–287
2. Hansen MC (1967) Three dimensional solubility parameter and solvent diffusion coefficient. Importance in surface coating formulation
3. Diorazio LJ, Hose DRJ, Adlington NK (2016) Toward a More Holistic Framework for Solvent Selection. Org Process Res Dev 20:760–773. https://doi.org/10.1021/acs.oprd.6b00015
4. Abbot S HSP Basics
5. Abraham MH (1993) Scales of solute hydrogen-bonding: Their construction and application to physicochemical and biochemical processes. Chem Soc Rev 22:73–83. https://doi.org/10.1039/CS9932200073
6. Poole CF, Atapattu SN, Poole SK, Bell AK (2009) Determination of solute descriptors by chromatographic methods. Anal Chim Acta 652:32–53. https://doi.org/10.1016/j.aca.2009.04.038
7. Chung Y, Vermeire FH, Wu H, Walker PJ, Abraham MH, Green WH (2022) Group Contribution and Machine Learning Approaches to Predict Abraham Solute Parameters, Solvation Free Energy, and Solvation Enthalpy. J Chem Inf Model 62:433–446. https://doi.org/10.1021/acs.jcim.1c01103
